# Ten practices for successful study coding in research syntheses: Developing coding manuals and coding forms

**DOI:** 10.1017/rsm.2025.10019

**Published:** 2025-06-23

**Authors:** Gena Nelson, Sarah Quinn, Sean Grant, Shaina D. Trevino, Elizabeth Day, Maria Schweer-Collins, Hannah Carter, Peter Boedeker, Emily Tanner-Smith

**Affiliations:** 1 Center on Teaching and Learning, College of Education, University of Oregon, Eugene, OR, USA; 2 Special Education and Communication Sciences and Disorders, Eastern Michigan University, Ypsilanti, MI, USA; 3 HEDCO Institute for Evidence-Based Educational Practice, College of Education, University of Oregon, Eugene, OR, USA; 4 Department of Psychology, College of Liberal Arts and Sciences, Iowa State University, Ames, IA, USA; 5 Department of Teaching, Learning, and Community Engagement, College of Education, Boise State University, Boise, ID, USA; 6 Department of Education, Innovation and Technology, Baylor College of Medicine, Houston, TX, USA

**Keywords:** coding procedures, data extraction, meta-analysis, study coding, systematic review

## Abstract

Study coding is an essential component of the research synthesis process. Data extracted during study coding serve as a direct link between the included studies and the synthesis results, allowing reviewers to justify claims about the findings from a set of related studies. The purpose of this tutorial is to provide authors, particularly those new to research synthesis, with recommendations to develop study coding manuals and forms that result in efficient, high-quality data extraction. Each of the 10 easy-to-follow practices is supported with additional resources, examples, or non-examples to help authors develop high-quality study coding materials. With the increase in publication of meta-analyses in recent years across many disciplines, a primary goal of this article is to enhance the quality of study coding materials that authors develop.

## Highlights

### What is already known?

- Study coding is the process of extracting data from studies included in a research synthesis, and the data from the process serve as a direct link between the studies and the synthesis results. Study coding is an essential, and time-consuming, component of conducting high-quality research syntheses.

### What is new?

- We present 10 essential practices for conducting high-quality study coding with a focus on how to develop coding manuals and coding forms.

### Potential impact for RSM readers

- We expect this tutorial to be a useful tool for developing coding manuals and coding forms for all scholars, but particularly for graduate students and scholars who are new to conducting research synthesis.

## 1 Introduction

Research syntheses play an important role in the decision-making process for practitioners and policy makers across a broad range of disciplines.^1^ Given this role—along with the proliferation of researchers conducting research syntheses—it is critical that researchers take steps to ensure rigor in the research process and reporting of findings.^2^ We use “research synthesis” to collectively refer to systematic reviews, meta-analyses, and other types of reviews in which authors collect and synthesize information from primary studies answering the same research question(s). See Table 1 for a brief overview of other important terminology that we use in this tutorial.

**Table 1:** Terminology

Term	Definition
Code	Selected code from a set of code response options for an item

Code description	Definition of code response options under each item

Code response options	Ways the coders can respond to an item, may be pre-determined or open-ended

Coder	Trained research project staff member who is responsible for collecting or extracting data from included primary studies

Coding form	The organizational system in which coders record responses, sometimes referred to as data collection form or database

Coding manual	The document that outlines the variables coders will capture, with definitions and descriptions of the variables, as well as coding decision rules, sometimes referred to as coding protocol, coding scheme, data collection tool, or codebook

Item	Variable identified for data extraction

Record	Any citation (journal article, dissertation or thesis, conference presentation, technical report, or other related document) that reports information about a study, may contain more than one study

Study	Defined by a unique sample on which data are collected and analyzed

Although it is important to conduct all aspects of research synthesis with precision and fidelity, the process of coding information from primary studies during a research synthesis (often referred to as “data extraction”) directly impacts the conclusions and interpretations that researchers and practitioners can make. Coding the included primary studies requires considerable attention to detail using a reliable coding manual and corresponding coding forms. In addition, study coding is time consuming: experts estimate that 60% of the time it takes to conduct a research synthesis is dedicated to study coding.^2^ The data collected during the coding process serve as a direct link between the included studies and the synthesis results, providing justification for claims made about the body of evidence synthesized.^3^^–^ ^5^ Therefore, the process of developing study coding materials should not be rushed or taken lightly.

## 2 Rationale for this tutorial

The purpose of this tutorial is to provide authors—particularly those new to research synthesis—with a condensed set of practices for study coding in research syntheses. Several resources exist for general recommendations about study coding. For example, Chapter 5 of the Cochrane Handbook highlights the types of information that should be included (e.g., participants, intervention characteristics, outcome measures) and general steps to get started with developing a coding manual (e.g., align the coding manual to tables that need to be created for the final manuscripts).^4^ Cooper and colleagues’ *The Handbook of Research Synthesis and Meta-Analysis* also provides recommendations for the type of information to include in a coding manual, as well as tips for structuring the coding form.^6^ Other researchers and organizations have provided guidance on the coding process, including different approaches to coding and reporting study coding reliability.^7^^–^ ^9^ We aim to complement this high-level advice with practical, yet specific, practices that can help researchers (especially graduate students and those new to research synthesis) with *developing* items and code response options in coding manuals.

This tutorial originated from the authors’ collective experiences developing their own coding manuals as well as teaching graduate students, early career scholars, and colleagues how to develop coding manuals for research syntheses. In reviewing and providing feedback on coding manuals in their earliest stages of development, we have noticed that many scholars new to research synthesis make common missteps in developing coding manuals and completing study coding. Given the important role that study coding plays in research synthesis, we aimed to provide readers with a set of easy-to-follow tips to improve their study coding process.

The practices that we highlight in this tutorial may also improve the reproducibility of research synthesis findings, as previous researchers have reported that coders often make errors when extracting data from studies during the coding process for meta-analysis.^10^^,^ ^11^ Clear coding manuals may help reduce coding errors. Moreover, experts have also reported that published research syntheses have often failed to appropriately provide information about their coding procedures (e.g., details regarding the specific coding items and responses^12^^,^ ^13^). This tutorial therefore provides information about best practices in coding, in general, to support reproducibility of research synthesis results.

## 3 Developing coding manuals and coding forms

This tutorial highlights 10 practices (see Table 2), with a primary focus on how to *develop* the tools that authors use to conduct high quality research syntheses—coding manuals and coding forms. There are several steps that authors need to consider, from determining the focus of the study coding to sharing the coding manual after it is finalized. Although this tutorial presents practices that typically occur in the order in which we have presented them, readers should consider that the steps associated with study coding are not always linear. Even after completing one step, authors may have to return to an earlier step given the iterative nature of study coding. Our team currently conducts research related to educational and health outcomes for students in preschool through 12th grade; therefore, many of the examples we provide are situated in the context of conducting a research synthesis on an academic or behavioral intervention offered in educational settings. However, we have aimed to write this tutorial for authors in any discipline to use these practices in research syntheses on any topic.

**Table 2:** Ten practices for successful study coding in research syntheses

Practices for successful study coding		
1	Conceptualize and share	• Identify information of interest
	coding manual	• Consult guidelines and stakeholders
		• Consider multiple data sources

2	Create coding manual	• Items should be single-barreled
	items	• Determine the code response format for each item
		• Ensure code response options are mutually exclusive
		and mutually comprehensive
		• Avoid ambiguous response options
		• Establish the difference between *No*, *NA*, and *NR*

3	Organize the coding	• Organize items according to how information is
	manual and coding form	presented in studies
		• Consider the relational structure of the data

4	Select coding form	• Compare different software
	software	• Select and program software

5	Pilot coding manual and	• Pilot, discuss, and revise the coding manual and
	coding form	coding form
		• Identify needs for training
		• Prospectively sharing the finalized coding manual
		and coding form

6	Begin study coding	• Include at least two coders
		• Randomly assign studies to coders

7	Manage the study coding	• Set coding deadlines
	process	• Establish regular communication
		• Optimize the coding form
		• Assess data quality

8	Identify and reconcile	• Identify studies for double coding
	disagreements	• Discuss disagreements and determine the final code
		• Contact study authors for missing data

9	Prepare materials for	• Ensure accessibility of coding manual and coding
	sharing	form
		• Organize and share materials (metadata, persistent
		• identifiers, data structure)

10	Evaluate study coding	• Ensure the review meets established methodological
	process	expectations

In our experience, involving practitioners in the development and coding process can help ensure authors are capturing information that is highest priority for non-research audiences. For example, educators are keenly interested in how research applies to their own local context, so coding for participant characteristics or program implementation requirements (when reported) are of utmost importance.^14^ Although involving non-researchers in research syntheses may follow a formalized model (e.g., involving stakeholders in finalizing research questions or including them as coders), stakeholder engagement via informal conversations can also meaningfully inform the coding process.^15^^,^ ^16^ This “lighter touch” stakeholder involvement is often more feasible for researchers who want to involve stakeholder expertise but have less-developed networks of non-researchers and/or fewer resources for compensating stakeholders.

### 3.1 Conceptualize and share coding manual

Before authors begin to draft the coding manual, they must take some preparatory actions. To ensure the coding process will address the focus of the research synthesis, authors should identify the types of items that need to be included in the coding manual to answer the research question(s) and register the review, as well as determine prespecified steps for including and extracting information from multiple data sources with the same sample.

#### 3.1.1 Identifying information of interest

Prior to drafting the coding manual, authors must consider the overall purpose of their research synthesis. While authors are identifying information of interest, they must also consider their access to resources for conducting the synthesis. For example, an author who is a doctoral student performing a research synthesis for a class project may have access to fewer technological resources (e.g., subscription-based software for coding) and human resources (e.g., availability of coders) than a group of seasoned systematic reviewers who are supported by grant funding to conduct a research synthesis. In addition, the more items a coding manual has, the more hours of training and study coding will be required. Nonetheless, one benefit of gathering information on more items can be a more comprehensive dataset, potentially allowing authors to answer other research questions (including exploratory questions) in additional research syntheses and publications. Considering resources in conjunction with the overall purpose of the project is helpful for establishing the size and scope of the coding manual.

We recommend at a minimum that authors begin by developing a list of items that the coding manual will need to include for authors to (a) describe the included studies, (b) develop tables and figures, (c) code studies for risk of bias assessment, and (d) sufficiently answer the research questions posed in the research synthesis.^4^ Authors may use an established framework—such as the population, intervention, comparator, and outcome (PICO) framework used to determine eligibility criteria for primary studies—to define coding categories that are likely to ensure the gathering of many of the essential details to answer their research question(s), write their final report, and create any necessary tables.^17^ Authors can directly transfer their eligibility criteria to items, code response options, and code descriptions in their coding manual. Authors can then expand their coding manual by thinking about what other items are needed to comprehensively describe the included studies’ population, interventions, comparators, and outcomes. It is also helpful for review authors to develop a template of the tables they plan on including in the report of the completed research synthesis.^4^ Sometimes, information presented in tables is purely descriptive or not used in analyses (e.g., a summary of the included study population characteristics) and could be missed in the development of a coding manual if the author is only focused on developing items that are specific to answering the research question(s). Authors should also consider what supplemental tables or figures they will present and ensure that their coding manual is aligned with those materials.

#### 3.1.2 Consult guidelines and stakeholders

To develop items for study coding, authors should explore commonly used guidelines for reporting, risk of bias assessment, and methodological quality assessment for their general discipline. For example, the Preferred Reporting Items for Systematic Reviews and Meta-Analyses (PRISMA) Statement and Journal Article Reporting Standards (JARS) checklists are resources that authors may use to identify the types of items to include in a coding manual.^8^^,^ ^9^ PRISMA and JARS provide reporting standards for research syntheses that can be used to better understand the essential components that need to be coded and included in the write-up of a research synthesis. The Cochrane Collaboration also has several resources and open access tools related to risk of bias that authors can use or adapt to align with the types of studies in their synthesis.^18^^–^ ^20^ Authors should also consider using disciplinary-specific guidelines for their field. For instance, education researchers might consider the What Works Clearinghouse guidelines that focus on standards and procedures for assessing the methodological quality and internal validity of studies.^8^

We recommend that review authors engage various stakeholders while drafting the coding manual to determine the types of items they would be interested in learning more about on a particular topic. For example, if the research synthesis is focused on assessing the effectiveness of home-based interventions for young children, we recommend that authors have informal discussions with parents, service providers, staff at parent organizations, or family-centered businesses (e.g., libraries, museums) to identify salient items to include in a coding manual. Having informal discussions can shed light on current trends or challenges in practice that may not be familiar to researchers. In addition, if authors are new to their research area (e.g., graduate students), we suggest engaging additional experts in the field to review the coding manual and provide feedback about the items and codes.

#### 3.1.3 Consider multiple data sources

In many cases, information about the same sample of participants may be reported in two or more different reports (e.g., one dissertation and one peer-reviewed journal article report findings from the same sample; two conference proposals and one peer-reviewed journal article report findings from the same sample). Before coding studies, authors should prespecify what procedures they will use to handle multiple data sources, link and keep track of individual reports presenting findings from the same sample, and plan procedures for extracting data from multiple sources. Ideally, we recommend that authors code information about a study from all available reports on that participant sample, given that different reports from a study sample may provide unique information (e.g., one article may present primary impact analyses needed to estimate effect sizes whereas a separate article may present valuable contextual information about implementation). However, this approach may not be feasible due to project resource constraints, or in scenarios where the authors have strong rationale for prioritizing information reported in some reports over others. For instance, authors may decide to prioritize coding of information from the ‘main report’ of a study’s confirmatory analyses focused on primary outcomes (vs. supplemental reports focused on secondary outcomes, secondary follow-ups, or secondary exploratory analyses from the same sample). Given the range of approaches that might be used to handle multiple data sources, authors should therefore clearly identify which reports contributed information that was included in their research synthesis. In scenarios where studies report findings for overlapping or dependent effect sizes or participant samples, authors will need to track and document these dependencies carefully so that they can be attended to at the analysis stage.^21^^–^ ^24^ The issue of multiple data sources spans the entirety of a research synthesis from the literature search stage to the data sharing stage; therefore, we recommend that authors review specific guidance on this issue as described in Mayo-Wilson et al.^25^

### 3.2 Create coding manual items

**Figure 1:**
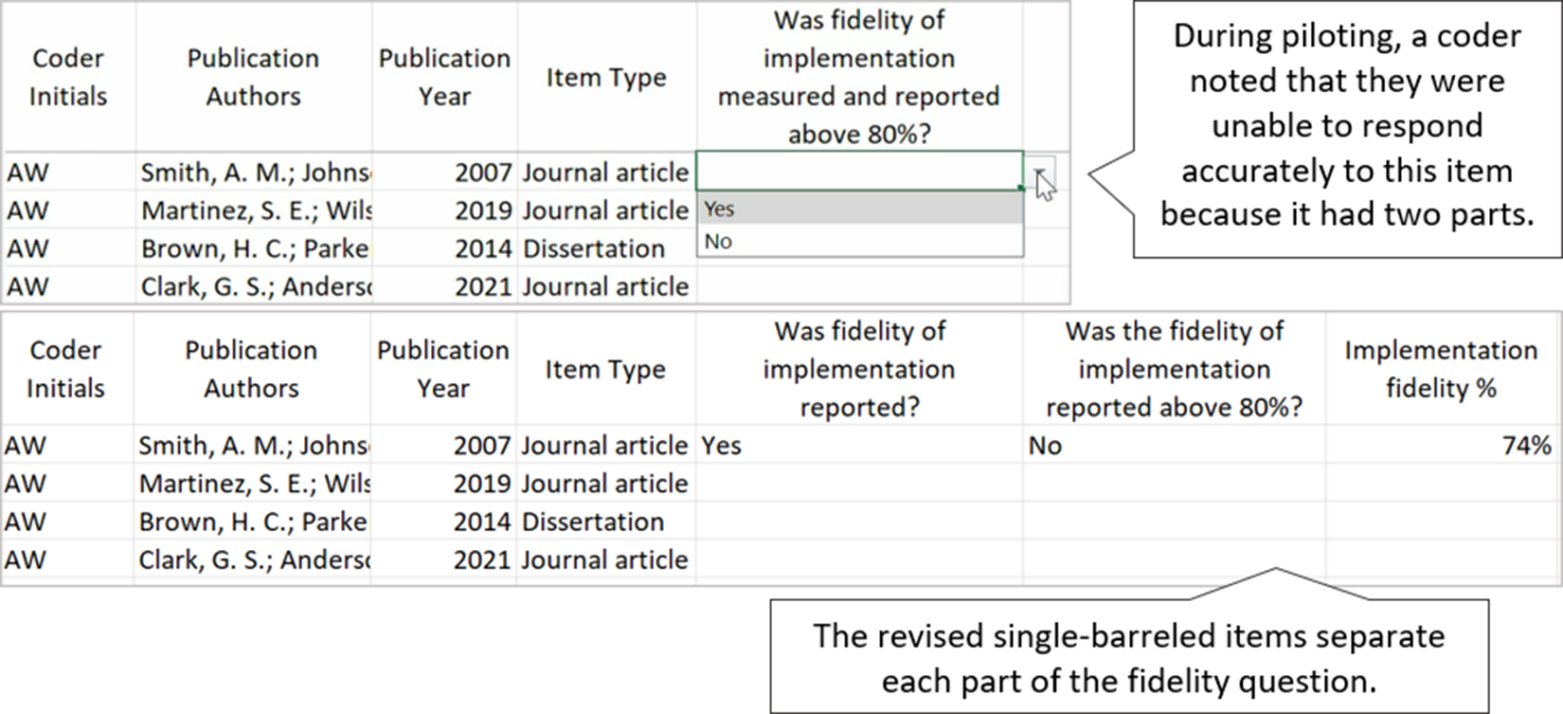
Codebook draft and revision: Ensure single-barreled items.

**Figure 2:**
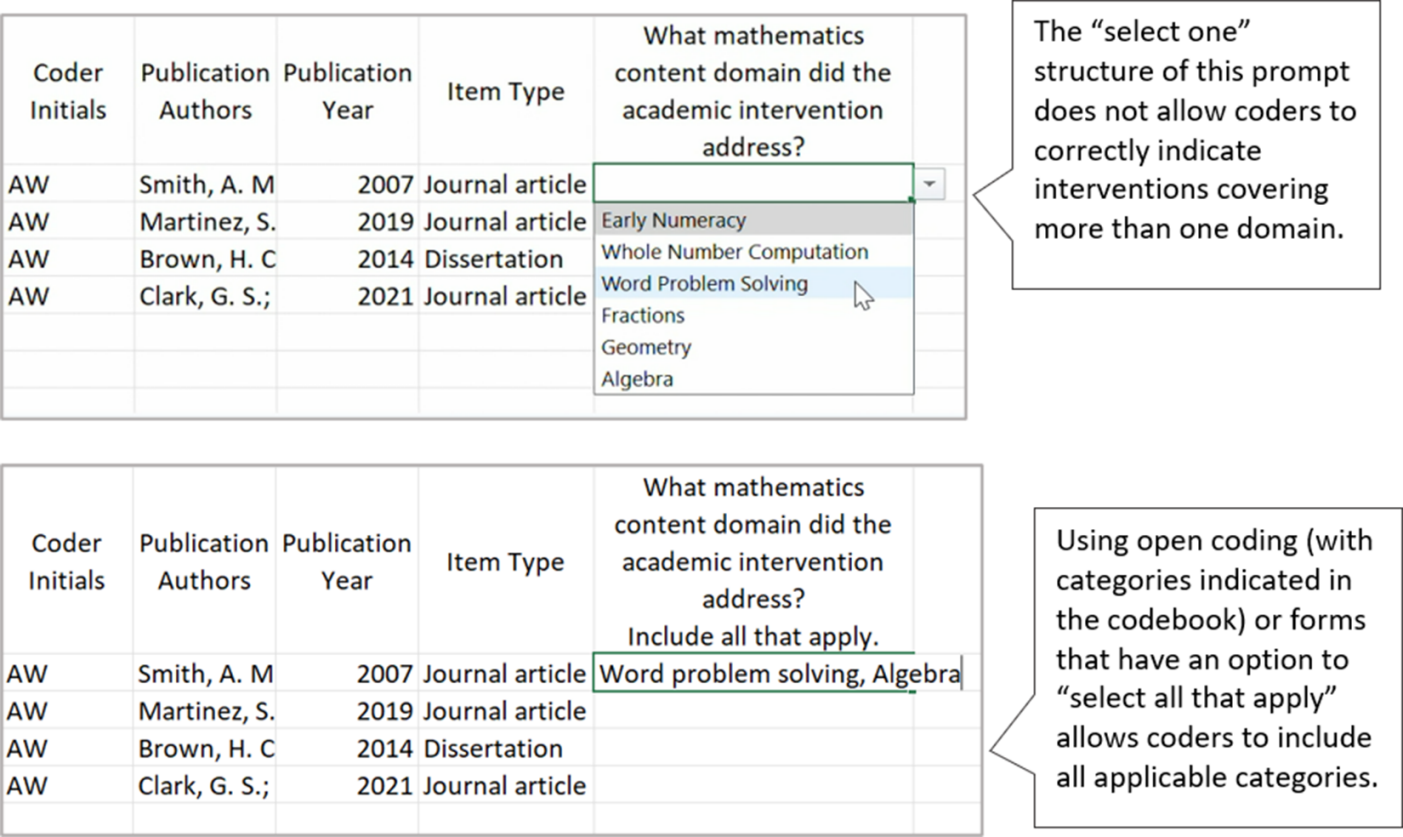
Codebook draft and revision: Choosing response formats.

**Figure 3:**
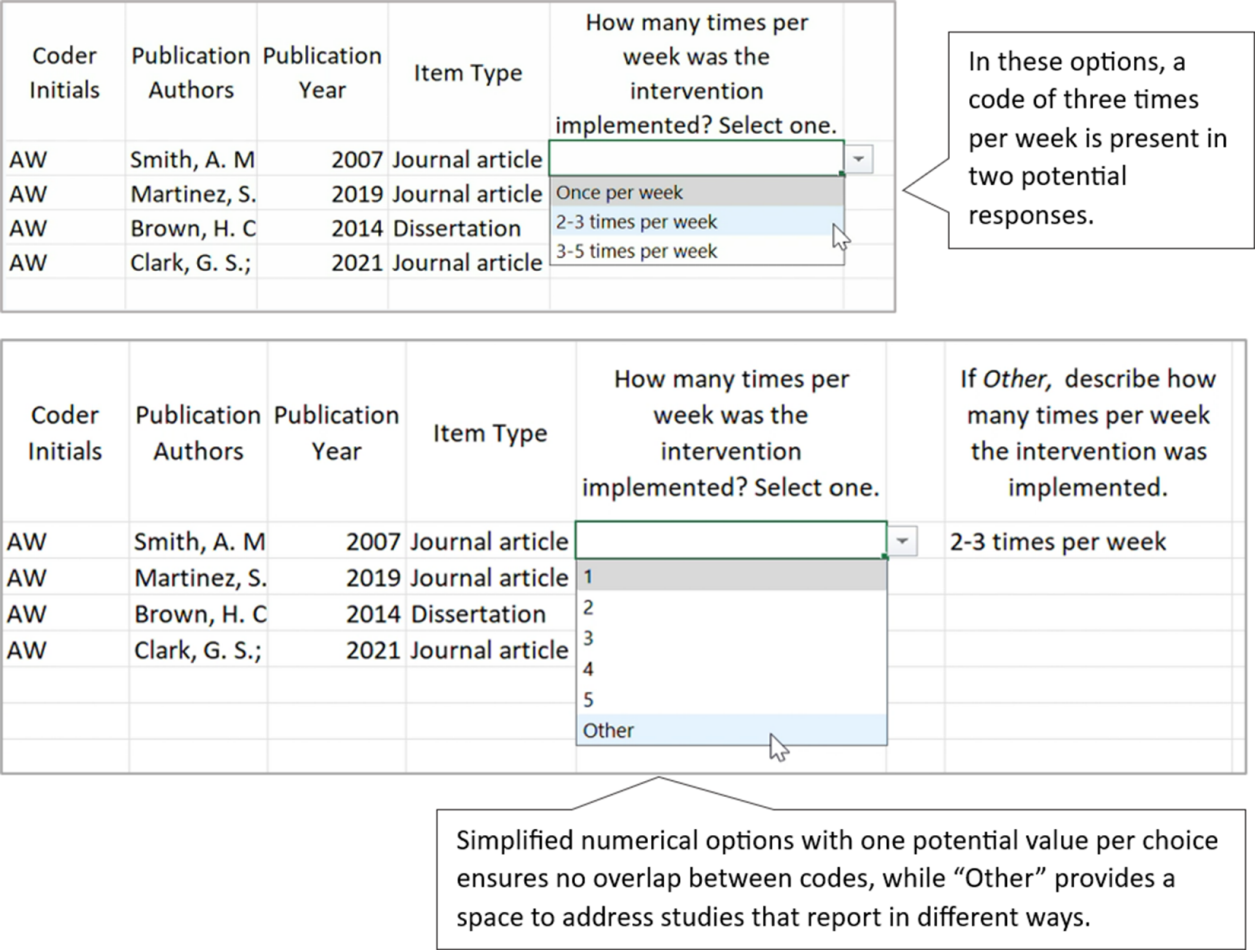
Codebook draft and revision: Ensure code response options are mutually exclusive and mutually comprehensive.

**Figure 4:**
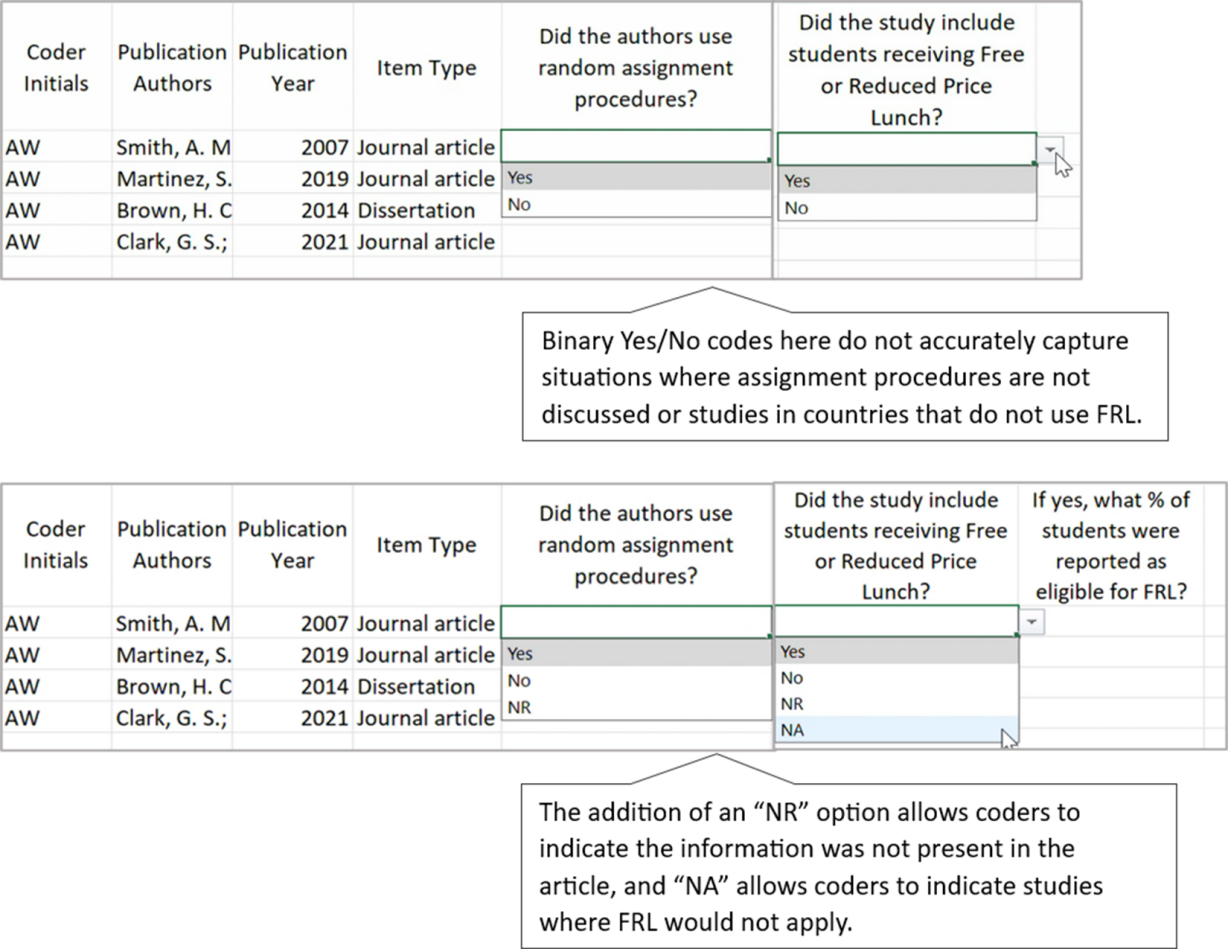
Codebook draft and revision: Establish the use of “No,” “NA,” and “NR.”

A well-constructed coding manual provides coders with all the information they need to make decisions during the coding process. When authors develop their coding manual, they should include clear item names, clear and concise code response options, and detailed code descriptions that are aligned with each code response option. Depending on the item type, the code descriptions may include definitions, a list of examples, potential non-examples, or other clarifying indicators. As authors develop and test their coding manual, we recommend they use single-barreled items, determine the most appropriate code response option format for each item, ensure code response options are mutually exclusive and mutually comprehensive, avoid ambiguous code response options, and establish the difference between similar code response options. Along with the descriptions below, we provide an example and non-example of several of these practices (see Figures 1–4).

#### 3.2.1 Items should be single-barreled

Items on the coding manual should be single-barreled, meaning that they require a coder to respond to or record one individual element of the study. For example, the item “Was fidelity of implementation measured and reported above 80%?” is not single-barreled because a coder must identify and record responses to two different components of fidelity of implementation, including “Was fidelity of implementation measured?” and “Was fidelity of implementation reported above 80%?” These two items should be separated in the coding manual.

#### 3.2.2 Determine the code response option format for each item

Authors will need to consider the best code response option for each item in the coding manual. Many items on a coding manual will be forced response, meaning that the coder is forced to choose one or more code response options (e.g., select “yes” or “no”; select 1 from a set of nominal codes; select all that apply). With items that are forced response, authors of the coding manual must also anticipate the structure of the code response options.^4^ Other items may allow coders to capture qualitative data in a free response format using text coding (e.g., an item that asks coders to “describe any services provided to the control condition”).

Authors will need to consider the code response option for each item separately because one response type (i.e., select one, select all that apply, open coding) cannot suit all items. For example, consider responding to the item “What mathematics content domain did the academic intervention address?” with the following code response options: early numeracy, whole number computation, word problem solving, fractions, geometry, and algebra. A “select one” code response option may not be an appropriate option for an intervention focused on word problem solving that includes computation of whole and rational numbers. In this case, a “select one” code response option forces coders to decide between two or more options that are all acceptable. This is likely to lead to unreliable responses between coders, as well as an incomplete data set related to this item. In this example, a code response option of “select all that apply” may be a better option.

Authors should also consider the tradeoffs associated with varying levels of detail related to the code responses for each item. For example, it may require more effort up front and during the coding process for coders to capture a specific code response across a set of items (e.g., separate binary yes or no codes for each grade level in school). This level of detail may not be needed for analyses in which authors plan to collapse grade levels across common grade bands (e.g., K-5, 6-8, 9-12) and may require more work at the data cleaning stage. However, if authors conduct detailed coding for items, their data may also be more accessible to future researchers who need or are interested in answering different research questions that require access to more specific code responses.

We recommend that authors also consider the advantages of open text coding for some items. Open text coding is particularly useful for items with too many possible response options to be accurately captured using a forced response list, for example, the study location or intervention characteristics. Open text coding also allows coders to expand on information already documented with forced response items, such as by providing a brief description after a forced response has been recorded (e.g., after selecting the grade levels included in the school, describe any other factors relevant to the school’s context), and attend to items that are included in the coding manual for descriptive purposes only. Additionally, open text coding can capture relevant information that may not have been considered at the time the coding manual was developed. For example, it can be difficult to know or anticipate all the outcome measures that may be used in a corpus of academic or behavioral intervention studies while developing the coding manual. Including an open text response option to briefly describe the outcomes measures that were noted as “other” in the forced response might be necessary. Text coding can also assign “summative, salient, or essence-capturing” information, which is not possible with forced response coding.^26^ This type of outcome may be particularly important to different stakeholders, who, for example, want to know about the context within which an intervention took place or how an outcome was measured.^27^ Indeed, open text coding may be particularly valuable in qualitative or mixed-methods research syntheses that aim to provide rich contextual information about a study’s implementation barriers and facilitators.

Although useful and often necessary, open text coding may pose some challenges, such as ensuring data can be enumerated for meta-analytic or other quantitative summaries. Further, reviewing the results of open text coding requires additional time to first assign codes, then categorize the coded data, and eventually identify themes. Establishing reliability for open text coding may also pose challenges for researchers, as it requires more exploratory and iterative coding behavior from coders, and after categories have been determined, coders often need to recode studies to validate the coding scheme.^7^ Thus, our recommendation is to minimize the amount of open text coding for analytic purposes and to reserve this type of coding for items that are exploratory or descriptive or considered high priority for stakeholders.

#### 3.2.3 Ensure code response options are mutually exclusive and mutually comprehensive

When items in the coding manual are forced response in which coders are required to “select one option,” authors should ensure that response options do not have any overlap. For example, if coders are responding to the item “How many times per week was the intervention implemented?” the following code response options have overlap: (a) once per week; (b) 2–3 times per week; (c) 3–5 times per week. In this example, there is overlap if the study reports that there were exactly 3 sessions per week (i.e., coders could select either option b or c and both would be correct). In this case, authors might consider developing code options without a range; 1 = one time per week; 2 = two times per week; 3 = three times per week; and so on. And of course, for any items planned for inclusion in quantitative analyses (e.g., meta-analyses), higher levels of measurement (i.e., interval or ratio measurement vs. nominal or ordinal) are always desirable, when possible.

Authors should also ensure that coding manuals with forced response options provide all possible responses. For example, studies may report the duration of an intervention as a range. In the example above where coders must “select one” a study with an intervention range reported as 2–3 times per week is not represented in the code response options. Therefore, we recommend that in many cases, authors also allow for a code of “other” to be selected. When coders select a code of “other” for any item, they should be required to add a qualitative explanation about why they used the code of “other” instead of the provided code response options.

#### 3.2.4 Avoid ambiguous code response options

Authors should avoid creating items with code response options that are descriptively ambiguous or not quantified. For example, code response options such as “none,” “some but not all,” “almost all,” and “all” are not quantified for coders. Two coders with the same training may interpret the use of “some but not all” and “almost all” in different ways that lead to an unreliable description of the results of the item. When possible, provide exact operational definitions for each of the code response options, or add quantifiers (e.g., a percentage that aligns to each code response option). When it is not possible to add quantifiers to code response options that are ambiguous, authors should consider adding a coder “inference level” item that indicates how confident (or not) they were when coding that item. See Wilson et al.^28^ for an example.

#### 3.2.5 Establish the difference between “no,” “not applicable,” and “not reported”

Our final recommendation for authors related to developing items on a coding manual is to establish clear guidance for coders on the difference between code response options of “no,” “not applicable (NA),” and “not reported (NR).” NR is important to include, especially when one of the code response options is also “No.” For example, consider the item, “Did the authors monitor the fidelity of the implementation delivery?” Providing coders with the code response options of “Yes” and “No” is insufficient. A study that does not report whether it monitored implementation fidelity should not automatically be recorded as “No,” because fidelity monitoring *may* have occurred but simply was not reported in the final manuscript. A code response option of NR, in this case, allows authors to determine if they need to contact authors about missing data that may be essential for the results of the research synthesis. In contrast, more straightforward items such as “Did the study include children in preschool?” are often appropriate for a binary “yes” or “no” code response options. In this case, if the authors report that all students were in kindergarten and first grade, a coder can reasonably assume that preschool children were not included in this study and indicate “no.” Authors of coding manuals must carefully consider the distinction between missing information and information that can be inferred.

Similarly, authors should consider how “NA” code response options represent information that is conditional or may not apply to a particular study. For example, a common socioeconomic status (SES) measure in education research syntheses, “What percentage of students were reported as being eligible for free or reduced-price lunch (FRL)?” is only applicable to studies conducted in the United States. Other countries use different indexes of SES to describe participants and FRL may not be relevant. Recording a code of “NR” for studies conducted outside of the United States for this type of item is not accurate. It is important that the coding manual clearly states when codes of “NA” should be used and provides clear instructions to the coder.

### 3.3 Organize the coding manual and coding form

As authors decide how information is presented in the coding manual and coding form, they should consider the ease with which a coder can read a study and simultaneously record information in the coding form. Authors should also consider that the structure of a coding form is dependent on the structure of the data being extracted.

#### 3.3.1 Organize items according to how information is presented in studies

We recommend that authors organize the coding manual and coding form in a manner that allows for efficient study coding by grouping items that are similar or likely to appear in the same part of the study together. For example, primary study authors typically report all participant demographic variables together, either in text or in a table; these items should therefore be presented together in the coding manual and coding form. If demographic items are spread throughout the coding form, it becomes time consuming for a coder to repeatedly leave and return to the same sections of a study multiple times to record information in the coding form. Likewise, when coding effect size data, means and standard deviations are often presented together so it is ideal to have these two statistics presented side-by-side in the coding form versus in separate sections. This strategy can also reduce errors in study coding.

#### 3.3.2 Consider the relational structure of the data

Authors will also have to consider the organization of the coding manual and coding form in terms of the structure of the data. Consider a research synthesis focused on social-emotional learning interventions. Some coding items may be related to student participants (e.g., sample size, demographics) and responses to these items may be reported in studies at the group level (e.g., intervention group, comparison group). Whereas other items, such as intervention features or study design, may be presented at the study level. Further, other items, such as characteristics of the outcome measures, may be coded at the level of the outcome measure or even at the effect size level. We recommend that authors separate areas of the coding form according to the different levels that information may be presented (e.g., study, participants, conditions, outcome measures, effect sizes). Separating the coding form in this manner may be more efficient for coders and lead to fewer coding errors; however, each part of the coding form must include the study’s unique identifier (and additional unique identifiers for each level of coding items in the relational data structure) to permit efficient merging of data during the analysis stage. Designing the coding form in this way will facilitate the creation of relational databases that are multifunctional and machine-readable.^29^

### 3.4 Select coding form software

Using software intended for conducting research syntheses can increase efficiency, functionality, and collaboration throughout the coding process and data analysis phase. There are many data collection tools for research syntheses, such as Covidence, DistillerSR, EPPI-Reviewer, MetaReviewer, Rayyan, and SRDR+.^30^^,^ ^31^ Microsoft Excel is a commonly used and familiar option for developing coding forms and is demonstrated in the figures presented in this tutorial. Other familiar tools might include Google Sheets, Microsoft Access, REDCap, or data files created within statistical software (e.g., R, SAS, and SPSS). Authors should select the software that meets the specific needs and resources of each research synthesis project. Software designed specifically for research syntheses (e.g., Covidence and DistillerSR) offer user-friendly coding functionality and are based on underlying relational databases; thus, these tools may automatically handle the relational structure of review data. Conversely, more generalist software tools (e.g., Microsoft Excel) typically offer simple, non-relational databases, and thus may require more effort by the review author to develop multiple coding sheets and unique identifiers can be appropriately merged and analyzed at the data analysis stage. If possible, it is therefore helpful to explore and pilot different options before committing to a specific software, although some may not offer a free trial.

#### 3.4.1 Compare different software

We recommend that authors compare different software based on their suitability to the research question and coding manual. For example, authors should take into account the types of data planned for collection and which stages of the review will be conducted with the software. Authors should look for specific features such as an easy-to-use interface for coders and project managers; collaboration features, such as assigning multiple coders; and customizable features, such as customized coding forms and data levels. Finally, authors should examine how the software organizes, presents, and exports data for use.^32^ Features like availability of product support may be important to consider based on the team’s familiarity and comfort level with the software.

#### 3.4.2 Select and program software

After reviewing different software, authors should decide on the software that fits most of their needs and begin programming their coding form into the software following the coding manual. When programming the coding form, make sure all item names and code response options are consistent with the coding manual. Adding some descriptive information from the coding manual to the coding form can help keep coders consistent and increase efficiency in the coding process. For example, authors might include an eligibility criteria table for easy reference. Maximizing the use of conditional items (i.e., coding fields that are only applicable and shown to coders when a specific criterial is met) can also greatly increase coding efficiency and reduce coder fatigue. Depending on the software, it may also be possible to program automated data collection fields that populate based on previous responses or create filters that control the flow of references through the project. It can be extremely helpful to review any user guides or product support information when programming to see what options exist for increasing efficiency in the coding process.

### 3.5 Pilot coding manual and coding form

Once authors have completely drafted their coding manual and coding form, we recommend they pilot the coding manual and coding form with members of their review team. Piloting the tools can serve multiple purposes, including identifying areas of the coding manual or coding form that need revision, identifying specific needs for training coders, and estimating the amount of time coders will need to code each study, which may be highly variable depending on the complexity and scope of the research synthesis. This point in the process is also a time to reconnect with stakeholders. While authors pilot the coding manual, they could ask a stakeholder to review it to identify any missing items or provide support in adding detail to the code descriptions. Connecting with stakeholders during coding manual piloting signals respect for stakeholder time (offering them a complete version to review rather than partial or unfinished versions they must review more than once) and allows for stakeholder input to contribute to changes before coding has begun.

#### 3.5.1 Pilot, discuss, and revise the coding manual and coding form

We recommend that authors pilot the coding manual and coding form with at least two studies, and when possible, select studies that report different types or amounts of information (e.g., select one peer-reviewed journal article and one dissertation; select one older study and one newer study that also provides supplemental files). When piloting, members of the review team should use studies that have been identified for inclusion in the current research synthesis or excluded studies that are closely aligned with the focus of the research synthesis. Piloting the tools for study coding allows the authors to identify any challenges with the coding manual or coding form. For example, during the pilot phase, coders may notice missing code response options or identify items needing additional details in the code descriptions. They may also identify missing items altogether and suggest additions to the coding manual. Coders may also identify challenges with the coding form, such as misalignment between the coding manual and coding form. We suggest that all members of the team (i.e., author(s) of the coding manual and coding form, and coders) meet to discuss potential edits and revisions. Meeting allows the authors of the coding manual and form to ask questions for clarification and gain additional understanding of any challenges with the tools. As final decisions are made, we recommend documenting changes to the coding manual and coding form and clearly communicating all changes to the research team (e.g., sharing a copy of the coding manual with final edits highlighted using track changes). After the meeting, the authors should review all comments and salient points from the discussion with the coders to make final decisions about the items, code response options, code descriptions, and coding form. Finally, we recommend that authors minimize changes to the coding manual after study coding begins.

#### 3.5.2 Identify needs for training

This stage of developing the coding manual and coding form also supports the primary author in identifying aspects of study coding that need additional emphasis or practice during training. Authors can determine which aspects of the coding manual and coding form were most challenging for the coders and can share related resources with coders before training. At a minimum, we suggest that the coder training be led by one or more content area experts according to the review topic, and that training includes: providing coders with background knowledge about the content area of the review, providing a review of the coding manual and coding form, and allowing for coding practice. Similarly, to prevent misapplication, training in coding items related to risk of bias or study quality assessment should be provided by trained methodologists with experience in the tool.^33^ Training should also require that coders meet a minimum threshold of reliability with the coding manual and form. For more detailed procedures on training coders, we recommend reviewing Giesen and Roeser’s practices for establishing a team-based approach to coding data and Cooper et al.’s handbook (specifically Section 9.6 on training of coders^34^).^6^^,^ ^35^

#### 3.5.3 Prospectively sharing the finalized coding manual and coding form

Just as with other forms of research, an important step at this phase in a research synthesis project is to register the study and prospectively share the protocol and analysis plan.^36^^,^ ^37^ The version of the coding manual and coding forms finalized after piloting should be among the materials prospectively shared.^38^^,^ ^39^ Some research synthesis publishers (e.g., Campbell Systematic Reviews and Cochrane Systematic Reviews) require a prospectively registered protocol be published prior to the publication of the full research synthesis. However, researchers who are not publishing their completed reviews in these outlets should register their review elsewhere, such as PROSPERO or the Open Science Framework (OSF).^40^^–^ ^47^ Given the multitude of options for where to register their study materials, the choice may be driven by disciplinary norms and preferences of the review authors. It may be difficult to anticipate all of the scenarios across a corpus of studies that may impact the development of the coding manual and coding form. To balance this challenge with prospective registration, we recommend that authors describe any changes made to the protocol after registration (e.g., in a “differences between protocol and review” section of the final report). The information provided to amendments to the coding manual should include the specific change, the reason for the change or revision, and the stage at which the change was made.^9^

### 3.6 Begin study coding

To begin study coding, research teams should identify coders and have procedures in place to ensure studies are randomly assigned to coders.

#### 3.6.1 Include at least two coders

During study coding, ideally at least two independent coders should be trained and responsible for study coding.^48^ Larger projects may require more coders to avoid assigning any one coder too many studies; however, more coders may also lead to delays in all coders establishing reliability with the coding manual and coding form. Smaller projects (e.g., a doctoral thesis) should at minimum include one primary coder and, when possible, one secondary coder for the purpose of assessing the reliability of the coding process. While coding, we recommend that coders read the entire study and all supplemental materials associated with it; if a study presents findings from a given sample in multiple reports, we again recommend coders read all reports associated with that study. This ensures that the coders do not miss any information, including information presented in supplemental reports, or information buried deep in appendices or supplemental materials.

#### 3.6.2 Randomly assign studies to coders

Ideally, studies should be randomly assigned among coders, with the team leader making manual adjustments as necessary to handle any potential conflicts of interest. For example, if any of the coders are authors of the included studies, the team leader should adjust the random assignments to ensure that no coder receives their own records to code. The same suggestion applies to articles in which a coder may be a former student or collaborator with the first author of an included study. This process removes any potential bias of coders coding information that is known only to them, and was not published in the report, because they are an author of an included study or a close collaborator to the first author of an included study. For large teams, the number of studies assigned to coders should be balanced such that all combinations of coder pairs are as equal as possible. Alternatively, coders may be assigned parts of the coding manual to code studies based on their areas of expertise.^6^ This process could be particularly beneficial for coding items that are highly technical or require in-depth content knowledge. For example, graduate students who have expertise in social–emotional interventions may have content knowledge to support most study coding for items related to basic study details, the participants, and intervention features. However, some graduate students may not yet have training to understand the different ways a study will report effect size information or have sufficient knowledge about different methodological issues that need to be critically appraised as part of risk of bias assessments. With highly technical items, authors can separate the coding manual into sections according to areas of expertise and then randomly allocate coding assignments within each section.

### 3.7 Manage the study coding process

In addition to a clear and concise coding manual and coding form, the study coding process also requires a team leader to guide the research team.^2^ Team leaders should set study coding deadlines and establish procedures for regular communication. We also recommend the team leader revise the coding form based on coder feedback as needed, and perform data quality checks during the coding process.^2^^,^ ^35^

#### 3.7.1 Set coding deadlines

Rather than waiting to receive all study codes by one final deadline, we recommend the team leader create a schedule of due dates that requires coders to turn in codes for specific studies throughout the coding process. Depending on the size of the review, the team leader may collect codes for studies weekly or bi-weekly, or after a certain number of studies are coded, for example, every five studies. This requires that coders begin coding studies as close to the training as possible, rather than waiting several weeks, or even months, to begin coding. This will likely result in greater adherence to the coder training, coding procedures, and ultimately coding reliability. Setting coding deadlines also allows a team member to calculate reliability of the coding throughout the coding process (and provide additional training or corrections needed to maintain high reliability), rather than wait until the end of coding to calculate reliability (see Section 3.8.1). Moreover, setting intermediate deadlines can help maintain momentum for the overall task.^35^ Setting intermediate deadlines also allows the team leader to monitor questions from coders, identify and address misconceptions, and regularly communicate with the team.

#### 3.7.2 Establish regular communication

Regardless of the scope or size of the synthesis, the team leader should carefully manage the study coding process with regular and consistent communication.^35^ Even with a well-thought-out coding manual and coding form, there are likely to be unexpected challenges that arise during study coding. Coders may ask the team leader for clarification on different items or response options as these challenges arise. The team leader can then communicate with the team to ensure that the clarifying information is applied consistently by coders. We recommend the research team meet at least weekly or bi-weekly to update the team on coding progress, ask clarifying questions, and discuss challenging items. This, of course, must be carefully balanced with the requirement that all coding is done *independently* by coders. Although changes to the coding manual should be minimal after independent coding begins, any changes to the coding manual (e.g., additional detail or examples to support coders) should be discussed during these meetings and documented.

#### 3.7.3 Optimize the coding form

To improve the efficiency of study coding and coder engagement, team leaders should continually refine the coding form based on feedback from coders. While coding all studies, coders may encounter unexpected study-specific nuances that require special consideration or recognize patterns of reporting that are not reflected in how the original coding form was programmed. Coders should also be encouraged to provide feedback about the optimal presentation of information, such as how and where to place coding fields and whether additional definitions or examples would be helpful to include within the coding form. Team leaders can then incorporate coder feedback into the coding forms to make them more user-friendly, reduce the likelihood of errors and coder fatigue, and expedite the study coding process.

#### 3.7.4 Assess data quality

During the coding phase, team leaders can perform data quality checks to ensure the integrity and accuracy of the coded data and increase the efficiency of data analysis. Assessing data quality can also reveal unanticipated issues with how coders interpret and use the coding manual or coding form. The team leader should review the coded data to verify all fields, code response options, and missing data are populating correctly. This can be done by exporting the data to ensure that missing data codes show up appropriately and as distinct from skipped fields. Additionally, the team leader should confirm that the item names and code response options in the coding form and the data export are consistent with the coding manual. Last, by exporting the data, the team leader can verify that the data from all levels (e.g., study level, group level, effect size level) can be merged efficiently for meta-analytic data analysis.

### 3.8 Identify and reconcile disagreements

As data collection commences and progresses, the review team should have processes in place to determine the reliability of the coding and reconcile any disagreements among double-coded studies. At minimum, authors should track and report overall reliability of the coding process; however, if possible and resources allow it, authors can track agreement across pairs of coders and consider reporting reliability between each pair of coders.

#### 3.8.1 Identify studies for double-coding

Views differ in the research synthesis field on the number or percentage of studies that must be double-coded. Although double-coding of all studies is ideal, research synthesis methodologists appreciate that some reviews may not have the resources to do so (particularly for large reviews), or that some coding manuals may be sufficiently simple to allow single-coding after a piloting phase that establishes high inter-rater reliability.^49^^–^ ^51^ One common and acceptable approach to completing double-coding is for teams to examine reliability of the study coding process at the beginning of coding (such as while piloting the measure or after the initial coder training), and once all coding is completed, calculate reliability, and then reconcile disagreements.^7^ However, there may be instances when research teams want to consider a different approach to examining reliability and reconciling disagreements, such as with very large reviews or when several coders on the research team are new to study coding. In these instances, we recommend that research teams examine reliability during the coding process, such as bi-weekly. This approach allows reliability to be calculated and for disagreements to be reconciled in a manner that may lead to greater reliability of coder behavior throughout the study coding process. For example, through this process, coders may determine that an item with frequent disagreement is the result of a coder misunderstanding information in the coding manual. Instead of allowing a coder to code all studies with this misunderstanding, the team member leading study coding can re-train the coder on specific items. This approach requires more organization at the front end of a review to ensure that the studies for double-coding are identified early and that those studies are being coded at approximately the same time by both coders. In cases where it is not feasible to double-code all studies, we recommend that authors consider double-coding a random subset of essential items. For example, authors could double-code all studies for information required to calculate effect sizes as well as items related to pre-specified moderator variables of interest. Regardless of approach, when only a subset of studies is double-coded, these studies should be randomly identified, assigned to a second coder, and coded independently in duplicate.

#### 3.8.2 Discuss disagreements and determine the final code

For disagreements that appear to be an error by one coder, coders can likely resolve the disagreement easily with a brief conversation between the two coders. We recommend that coders assigned to the same study are provided with a list of disagreements prepared by another author (e.g., the lead coder, first author) and shared prior to the meeting. This gives coders time to review the items with discrepancies and their own original codes. Coders can then determine if the coding discrepancy was due to an error, such as accidentally selecting the wrong code, or if they missed information in the study report. After coders have had a chance to review the discrepancies, then they should meet to discuss the disagreements, and one coder should be the record keeper of the final coding decision that is returned to the lead coder. For disagreements that cannot be easily resolved, we recommend that a third team member participate in a discussion about the disagreement to determine the final code.^4^ We also recommend that team leaders be mindful of any power differentials among team members that could cause one coder to be overly submissive/assertive during coding disagreement discussions (e.g., one coder is a graduate student and another is a postdoctoral scientist). Ideally, during team trainings and meetings, the team leader should explicitly discuss and actively model a team culture that values and rewards healthy discussion and debate.

#### 3.8.3 Contact study authors for missing data

After determining the final code, coders should determine if there is any essential information missing from the published report. For example, missing information to calculate effect sizes (e.g., sample size) or specific moderator variables of interest. If there is missing essential information, coders could contact study authors to request this information. When contacting authors, we recommend the coder minimize burden of this request by including a list of missing information for the study author to respond with or a template for them to fill out. We also recommend including the specific report with missing information attached to the request to both increase the chances that the author will reply and decrease the chance the author sends information for the wrong published report. Finally, we recommend providing study authors with a clear deadline for their response, providing a reasonably appropriate timeframe to respond (i.e., at least 2 weeks). See Pigott and Polanin^2^ for more information about and options for handling missing data.

### 3.9 Prepare materials for sharing

Sharing the coding manual and coding form on an open access platform is crucial for promoting transparency and reproducibility of review findings.^52^ When preparing materials for sharing, the review authors should ensure they are accessible and clearly organized.

#### 3.9.1 Ensure accessibility of coding manual and coding form

Prior to sharing, authors should review the materials and make them as user-friendly as possible. For example, the coding manual should be shared in a standard, accessible format such as a PDF and arranged clearly with section and question headers. Providing comprehensive metadata on the coding form (e.g., item names and question descriptions) in a concise format, such as Excel, makes it easier for the user to understand the data collected on the coding form. Authors should also consider creating a detailed README file to increase the accessibility of materials. This file should explain the purpose of the review, provide a clear explanation of each material file and brief description of what information the file provides, and give step-by-step instructions to navigate through the review process and corresponding files. Consistent question headers and item names should be used throughout all materials.

#### 3.9.2 Organize and share materials (metadata, persistent identifiers, data structure)

After the research synthesis materials are prepared for sharing, the author must select an appropriate open access platform to host their materials. Repositories like the OSF that assign persistent identifiers, such as DOIs, allow for long-term availability and provide a consistent citation over time. Domain-specific or disciplinary-specific repositories can also increase the visibility of the materials to relevant researchers. Once a repository is selected, the accessible review materials should be clearly organized within the repository (e.g., grouping related documents, clear file names and structure). The README file should be the first document the user sees when accessing the repository; it should include all information needed for a user to find and understand what is in each file. All project files (including but not limited to the coding manual and coding form) should be included in the repository and corresponding README file.

### 3.10 Evaluate study coding process

As a final step, we recommend that review authors evaluate their study coding process to ensure that a given review meets established methodological expectations for research syntheses, and to promote a continuous quality improvement process among teams that have a program of research conducting research syntheses.^49^^,^ ^51^ For example, efforts should have been made to minimize error in data collection, such as a structured data extraction form that went through a piloting process.^50^ In addition, ideally the review involved at least two independent assessors performing data collection with a consensus process when disagreements arose; this process could involve either data collection in duplicate for all included studies, for a sub-sample with sufficient reliability (e.g., a kappa score of 0.80 or greater), or with the second review reading the article in detail to both check collected data for accuracy and ensure that no relevant information was missed.^49^^,^ ^50^ Lastly, authors should check whether sufficient study characteristics were collected for readers to be able to interpret the results, investigate heterogeneity, and consider applicability to their contexts of interest.^50^

## 4 Conclusion

The purpose of this article was to highlight 10 practices for successful study coding for research syntheses, specifically related to the development of coding manuals and coding forms to ensure high-quality, reliable data extraction. These practices aim to ensure a successful study coding process that supports authors in drawing conclusions from an extant body of literature. Future research is needed to empirically evaluate the impact of these practices on reliability and coding quality, yet we believe that the research community will benefit from these practices, and their use will result in stronger research syntheses. The 10 practices begin with developing a strong focus and plan for eventual reporting; proceed through the stages of creating and organizing well-designed coding manuals and coding forms, piloting the coding tools, managing the coding process, and reconciling disagreements; and conclude with preparing the coding materials for sharing and evaluating the data collection process. Each of these steps contains recommendations to address common challenges and errors in the coding process. As stakeholders and policymakers look to research syntheses to make decisions and draw conclusions about evidence-based practice, researchers have a responsibility to present the most accurate data and synthesized conclusions drawn from a body of literature. High-quality coding strategies are a critical component of ensuring the research synthesis process is efficient, reliable, and replicable; and the practices presented here will support researchers in the development of such strategies. Readers can find high-quality examples of coding manuals on various open access platforms, OSF or other institutional repositories.^53^

## Data Availability

Data sharing is not applicable to this article as no new data were created or analyzed in this study.
